# Photochemical reactions of biomass derived platform chemicals

**DOI:** 10.3389/fchem.2024.1485354

**Published:** 2024-12-10

**Authors:** Norbert Hoffmann, Mario Andrés Gomez Fernandez, Arthur Desvals, Corentin Lefebvre, Clément Michelin, Mohammed Latrache

**Affiliations:** ^1^ Institut de Physique et de Chimie des Matériaux de Strasbourg (IPCMS), CNRS, Université de Strasbourg, UMR 7504, Strasbourg, France; ^2^ Laboratoire de Glycochimie et des Agroressources d’Amiens (LG2A), Université de Picardie Jules Verne (UPJV), Amiens, France; ^3^ Université Clermont Auvergne, Clermont Auvergne INP, CNRS, ICCF, Clermont-Ferrand, France; ^4^ Biomolécules: Conception, Isolement et Synthèse (BioCIS), UMR CNRS 8076, Université Paris-Saclay, Orsay, France

**Keywords:** carbohydrates, furans, levoglucosenone, lignin, organic synthesis, photocatalysis, terpenes, vanillin

## Abstract

Platform chemicals obtained from biomass will play an important role in chemical industry. Already existing compounds or not yet established chemicals are produced from this renewable feedstock. Using photochemical reactions as sustainable method for the conversion of matter furthermore permits to develop processes that are interesting from the ecological and economical point of view. Furans or levoglucosenone are thus obtained from carbohydrate containing biomass. Photochemical rearrangements, photooxygenation reactions or photocatalytic radical reactions can be carried out with such compounds. Also, sugars such pentoses or hexoses can be more easily transformed into heterocyclic target compounds when such photochemical reactions are used. Lignin is an important source for aromatic compounds such as vanillin. Photocycloaddition of these compounds with alkenes or the use light supported multicomponent reactions yield interesting target molecules. Dyes, surfactants or compounds possessing a high degree of molecular diversity and complexity have been synthesized with photochemical key steps. Alkenes as platform chemicals are also produced by fermentation processes, for example, with cyanobacteria using biological photosynthesis. Such alkenes as well as terpenes may further be transformed in photochemical reactions yielding, for example, precursors of jet fuels.

## Introduction

Sustainability play a key role for the development of mankind. In the case of chemical industry, this has been recognized very early. More than 100 years ago, G. Ciamician has published ideas about a non-polluting chemical industry based on photochemical and enzymatic reactions for the production of biomass as it is done by green plants (Ciamician, 1912; Ciamician, 1908). In fact biological organisms using photosynthesis constitute the biggest chemical industry with an annual production of 1.7 ∙ 10^11^ t per year (Lichtenthaler and Peters, 2004). Lignocellulose represents the major part of biomass (Shinde et al., 2020). It is mainly composed of carbohydrates (C_6_ sugar based material such as celluloses or starch and C_5_ sugar based material such as hemicelluloses) and on lignin which is an important source of aromatic compounds and an important source of platform chemicals. Various criteria for sustainable or green chemistry have been defined (Anastas and Kirchhoff, 2002). Approaching most closely the methods of chemical production to those used by nature is one of the strategies for a sustainable chemical industry. Another one is the optimization of already existing processes in view to reduce the environmental impact. This can be done, for example, by diminishing waste formation, simplifying the production processes by reducing the number of steps in multi-step syntheses or by using renewable feedstock.

In this regard, biomass as renewable feedstock play an important role. The molecular structure of biomass is different form corresponding fossil carbon compounds (Shinde et al., 2020; Wertz and Bédué, 2013; Barrault et al., 2018; Marion et al., 2017; Ravelli and Samorì, 2021; Gallezot P., 2012; Behr and Seidensticker, 2018). For this reason, transformations or production processes can be carried out and multi-step syntheses can be simplified which makes them more competitive form the economic and ecological point of view (Tietze, 1996; Groß et al., 2020). In the context of academic research, a lot of syntheses have been published with much more than 25 steps. Especially in the context of an industrial application and the environmental impact, these research approaches have been criticized (Tietze et al., 2000; Hudlicky, 1996; Tietze and Tietze, 2014). For example, when oxygen rich compounds are needed, they should preferentially be produced from carbohydrates because this renewable feedstock is oxygen rich (Lichtenthaler and Peters, 2004; Lichtenthaler et al., 2006) and the number of oxidation steps can be diminished (Levy and Fügedi, 2006). In some corresponding multi-step syntheses with fossil platform chemicals, complex chemo-, regio- or stereoselective oxidations are involved which makes them less competitive. In general, platform chemicals are key elements of the chemical industry as far as the production of bulk products or fine chemicals is concerned. The transformation of such compounds originating from biomass under sustainable conditions is therefore significant for the development of the chemical industry (Sheldon, 2014; Farmer et al., 2015; Shinde et al., 2020; Arias et al., 2020).

In the same context, organic photochemical reactions may be discussed. Using such reactions, compounds or compound families can be produced that are not or difficultly available by more conventional methods of organic synthesis (Turro and Schuster, 1975; Hoffmann, 2008; Bach and Hehn, 2011; Kärkäs et al., 2016a; Liu and Li, 2017; Zhu et al., 2024). This behavior is explained by the fact that photochemical excitation changes the electronic configuration of a molecule (Klán and Wirz, 2009). Many photochemical reactions are carried out without additional chemical reagents and activation of the starting compound occurs only by absorption of a photon. In this context, the photon is considered as a traceless reagent (Hoffmann, 2012; Oelgemöller et al., 2007) These reactions now gain in interest in the chemical industry (André et al., 1992; Braun et al., 1991; Bonfield et al., 2020; Moschetta et al., 2024). Recent activities in the domain of chemical engineering of photochemical reactions favor this interest (Elliott et al., 2014; Oelgemöller, 2014; Loubière et al., 2016; Noël, 2017; Zondag et al., 2023) In the context, of sustainable chemistry, it should also be mentioned that photochemical reactions can be carried out with sunlight as a renewable energy source (Oelgemöller, 2016). Some of such procedures are interesting in the context of an industrial application. Organic photochemistry has recently experienced a rebirth due to a wide range of work with different kinds of photocatalysis (Michelin and Hoffmann, 2018a; Michelin and Hoffmann, 2018b), especially photoredox catalysis applied to organic synthesis must be mentioned here (König, 2020; Stephenson et al., 2018; Marzo et al., 2018; Nicholls et al., 2016). Photochemical reactions are also studied in the context of depolymerization of biomass (Ouyang et al., 2022; Wu et al., 2020; Chen et al., 2021; Rao et al., 2021; Nwosu et al., 2021).

Both approaches, the transformations of biomass or biomass derived chemicals and the application of photochemical reactions significantly extend the space of chemical structures (Gómez Fernández and Hoffmann, 2023). The combination of these approaches also opens perspectives for a sustainable chemical industry. The present review deals with typical photochemical transformations of corresponding platform chemicals. The production of novel compounds is particularly focused.

## Platform chemicals from carbohydrates

**SCHEME 1:**
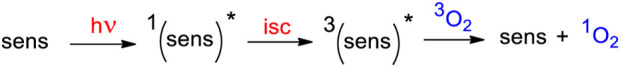
Generation of singlet oxygen by triplet sensitization.

Carbohydrates of carbohydrate based biopolymers are an important source of furans and many other compounds (Guigo et al., 2021; Mika et al., 2018). Furan compounds undergo easily photooxygenation involving singlet oxygen (Gollnick and Griesbeck, 1985; Montagnon et al., 2014; Montagnon et al., 2016). Among various methods (Nardello-Rataj et al., 2016), the photochemical production of singlet oxygen is a particularly attractive one (Ghogare and Greer, 2016; Bartoschek et al., 2005). In this case, the singlet species (Minaev, 2007; Schweitzer and Schmidt, 2003; Mittal et al., 2020) is generated form triplet oxygen by sensitization (Scheme 1). After photochemical excitation to the singlet state, the sensitizer (sens) undergoes intersystem crossing (isc) to the triplet state. Possessing the same spin multiplicity as oxygen at its ground state an interaction of both species is spin allowed. The sensitizer returns to its singlet ground state while the oxygen is excited to its singlet state. As the singlet energy of oxygen is relatively low (23 kcal mol^−1^), a large variety of sensitizers are used such as almost all kinds of dyes, organic and inorganic semiconductors, coordination compounds or nanoparticles with corresponding properties.

**SCHEME 2:**
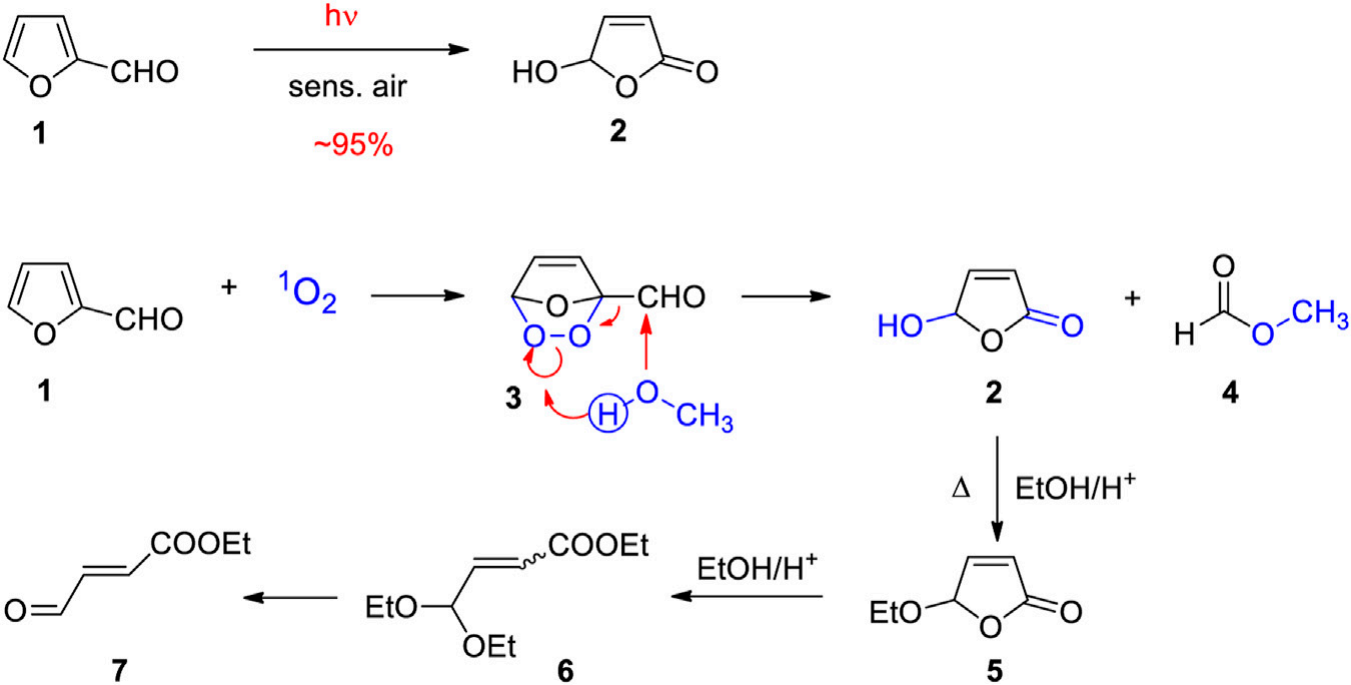
Photooxygenation of furfural **1** yields hydroxyfuranone **2** and related derivatives.

Furfural **1** is a furan derivative that is easily obtained from pentoses or hemicelluloses by dehydration (Zeitsch, 2000; Kabbour et al., 2020; Martel et al., 2010; Jaswal et al., 2022). The photooxygentation of this compound is very efficient and yields 5-hydroxy-2(5H)-furanone **2** (Scheme 2) (Schenck, 1953). The reaction starts with the addition of singlet oxygen leading to the endo peroxide **3** (Cottier et al., 1986). The reaction is often carried out with alcohols as solvent, in particular methanol or ethanol which attacks the endo peroxide intermediate **3** at the aldehyde function. Hydroxyfuranone **2** is generated by release of a corresponding formic ester **4**.5-hydroxy-2(5H)-furanone **2** is also a platform chemical (Esser et al., 1994; Badovskaja et al., 2021; Palai et al., 2024). It is easily transformed into corresponding 5-alcoxy-2(5H)-furanones like **5** or acyclic compounds such as **6** or **7** that are flexible synthesis intermediates (Scharf and Janus, 1978). Hydroxyfuranone **2** was used, for example, in asymmetric synthesis (Marinković et al., 2004; Feringa and de Jong, 1992; Moradei and Paquette, 2003; Riguet, 2011). In this context, studies on chiral induction in reactions of such furanones at their excited state should be mentioned. Due to photochemical excitation, the structures changes which also modify steric hindrance and relevant stereoelectronic effects (Marinković et al., 2004; Hoffmann et al., 1994; Hoffmann and Scharf, 1991; Bertrand et al., 1998; Fréneau et al., 2016).

**SCHEME 3:**
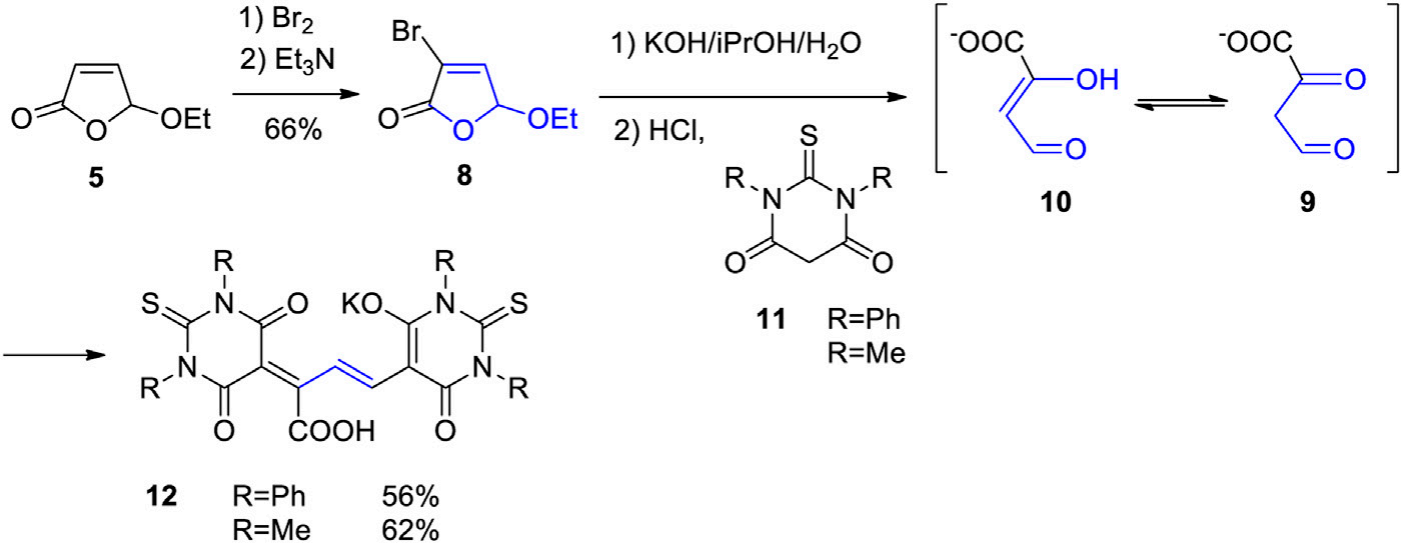
Synthesis of polymethine dyes of oxonol type starting with ethoxyfuranone **5** obtained from hydroxyfuranone **2**.

The photooxygenation of furfural can be carried out on large scale in the laboratory. Recently, an experimental procedure for the transformation of 100 g in 1.5 L of ethanol has been reported in detail (Desvals et al., 2022). Large scale transformations for 40-L-solutions have been carried out using sunlight (Esser et al., 1994). As a recent example of an application to organic synthesis, ethoxyfuranone **5** was transformed into a polymethine dye (Scheme 3) (Desvals et al., 2022). In the presence of bromine, the α-bromo derivative **8** is formed which leads to an increase of the oxidation state in this position. Thus hydrolysis yields the malondialdehyde intermediate **9** or its tautomer **10**. Condensation with thiobarbituric acid derivatives such as **11** yields oxonol dyes **12**. The present synthesis enabled a physico-chemical characterization of such dyes. These dyes play an important role in the photometric detection and quantification of enzyme activities (Unger, 1981; Nakashima et al., 1983) such as pectinlyase (Nedjma et al., 2001). In such tests, the intermediates **9** and **10** are generated from corresponding metabolites.

Further applications to the synthesis of biodegradable surfactants have been reported (Gassama et al., 2013; Gassama et al., 2009; Yue and Queneau, 2022). Furanone derivatives are interesting monomers for radical polymerization (Le Dot et al., 2024). Nevertheless, they undergo difficultly homo-polymerization. It was shown that copolymerization of alcoxyfuranones with electron rich monomers such as enolethers is very efficient (Poskonin et al., 1999; Lepage et al., 2023). The addition of photochemically generated radicals (Hoffmann, 1994; Bertrand et al., 2000a; Bertrand et al., 2000b; Harakat et al., 2006; Hoffmann et al., 2006) to furanones using ketones as sensitizer has been carried in continuous flow reactors (Yavorskyy et al., 2012; Yavorskyy et al., 2011) or microreactors (Shvydkiv et al., 2010). This reaction is suitable for the evaluation of different kinds of these reactors (Shvydkiv et al., 2011; Oelgemöller et al., 2014). Similar reactions have been carried out with inorganic semi-conductors as sensitizer (Marinković and Hoffmann, 2001; Marinković and Hoffmann, 2003; Marinković and Hoffmann, 2004).

**SCHEME 4:**
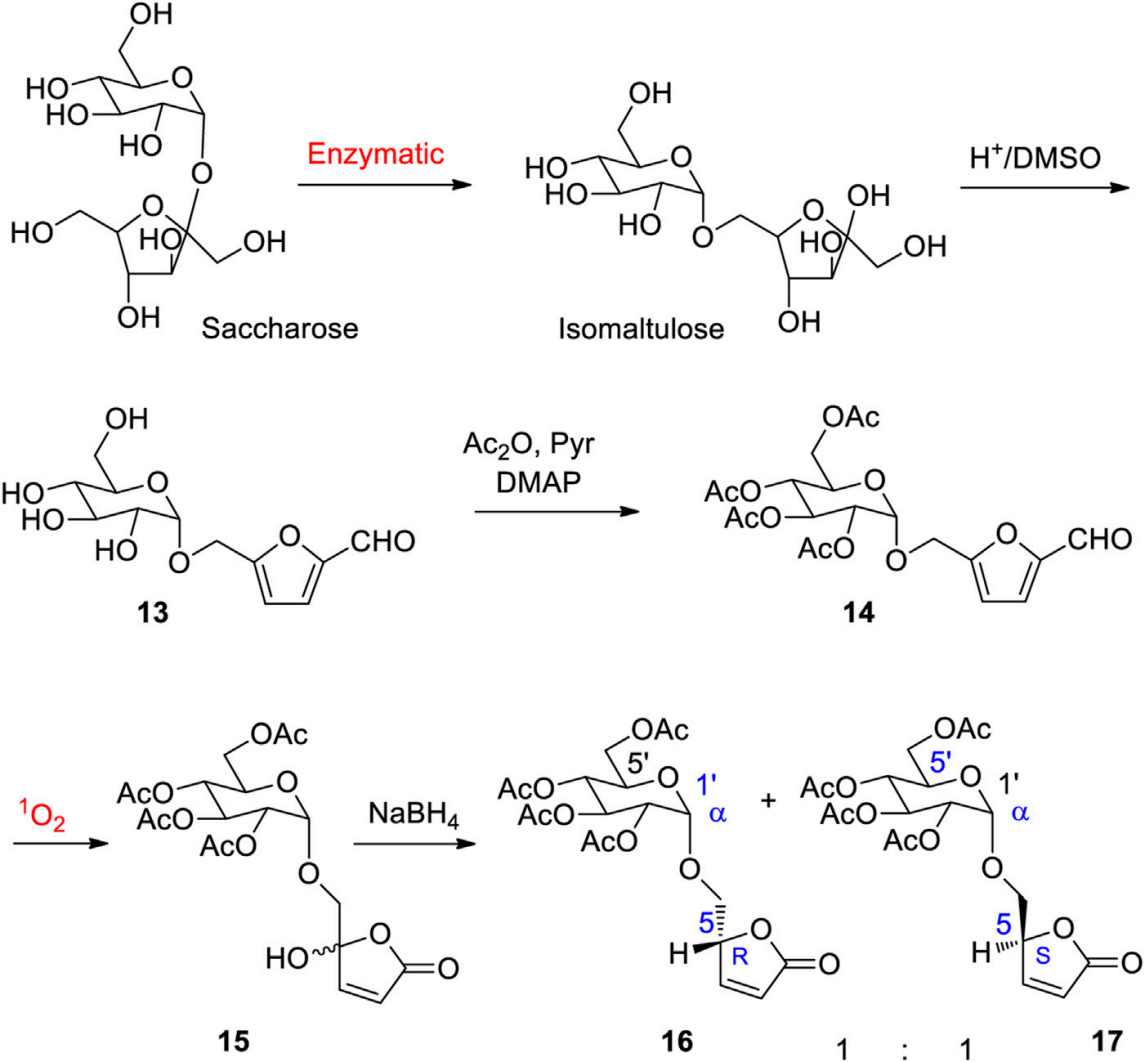
Selective dehydration of a furanosyl moiety in the presence of a pyranosyl group in isomaltulose. Photooxygenation of the furan substituent.

As mentioned in previous paragraphs furans are obtained by dehydration of carbohydrates (Takkellapati et al., 2018). The efficiency of this process depends on the structure of the sugar precursors. A corresponding dehydration sequence is efficient when furanoses react, partly because these compounds contain the five membered ring of furans. The equilibrium between a pyranose and furanose form must be shifted to the furanose. Glucose is a major element of biomass and its transformation by dehydration into the corresponding hydroxymethylfurfural (HMF) and corresponding derivatives such as 2,5-furandicarboxylic acid or 2,5-diformylfuran is of high interest (for selected reviews see: Yue and Queneau, 2022; van Putten et al., 2013; Hou et al., 2021; Li, 2023; Post et al., 2023; de Vries, 2017; Al Ghatta and Hallett, 2023; Zhang S. et al., 2023; Velty et al., 2022; Shinde and Rode, 2020; Lewkowski, 2001). The selective dehydration of a furanose moiety in the presence of a pyranose structure has well been performed in the case of isomaltulose (Scheme 4) (Lichtenthaler et al., 1993). This disaccharide is produced by enzymatic isomerization of saccharose (Hagen and Lorenz, 1957). The dehydration of isomaltulose yields Glucosylmethylfurfural (GMF) **13**. This compound is an interesting synthesis intermediate for the preparation of numerous bioinspired molecular structures (Tan et al., 2015). It should be pointed out that such α-annomeric derivatives of glucose are difficultly available by conventional synthesis techniques of carbohydrate chemistry (Levy and Fügedi, 2006). For different proposes, protecting groups can be introduced (**14**). In this case, the photooxygenation under conditions previously described for the transformation of furfural yields two epimers of hydroxyfuranone **15**. After reduction, the two furanone derivatives **16** and **17** have been obtained (Jahjah et al., 2010). In the present case, a study on stereoelectronic effect in photochemically induced hydrogen atom transfer reactions (HAT) (Jahjah et al., 2010; Hoffmann, 2016; Hoffmann, 2015; Hoffmann, 2017) was carried out. The photooxydation conditions are compatible with the presence of a variety of functional groups. Thus 5-(azidomethyl)furfural was transformed with a similar reaction sequence into 5-aminolevolinic acid hydrochlorid that is a natural herbicide (Mascal and Dutta, 2011). Hydroxymethyl furanones also called hydroxymethyl butenolides are also valuable synthons for a broader application to organic synthesis (Flourat et al., 2020). Photooxygenation processes at the industrial scale are well known (Rojahn and Warnecke, 1980; Turconi et al., 2014; Wau et al., 2021).

**SCHEME 5:**
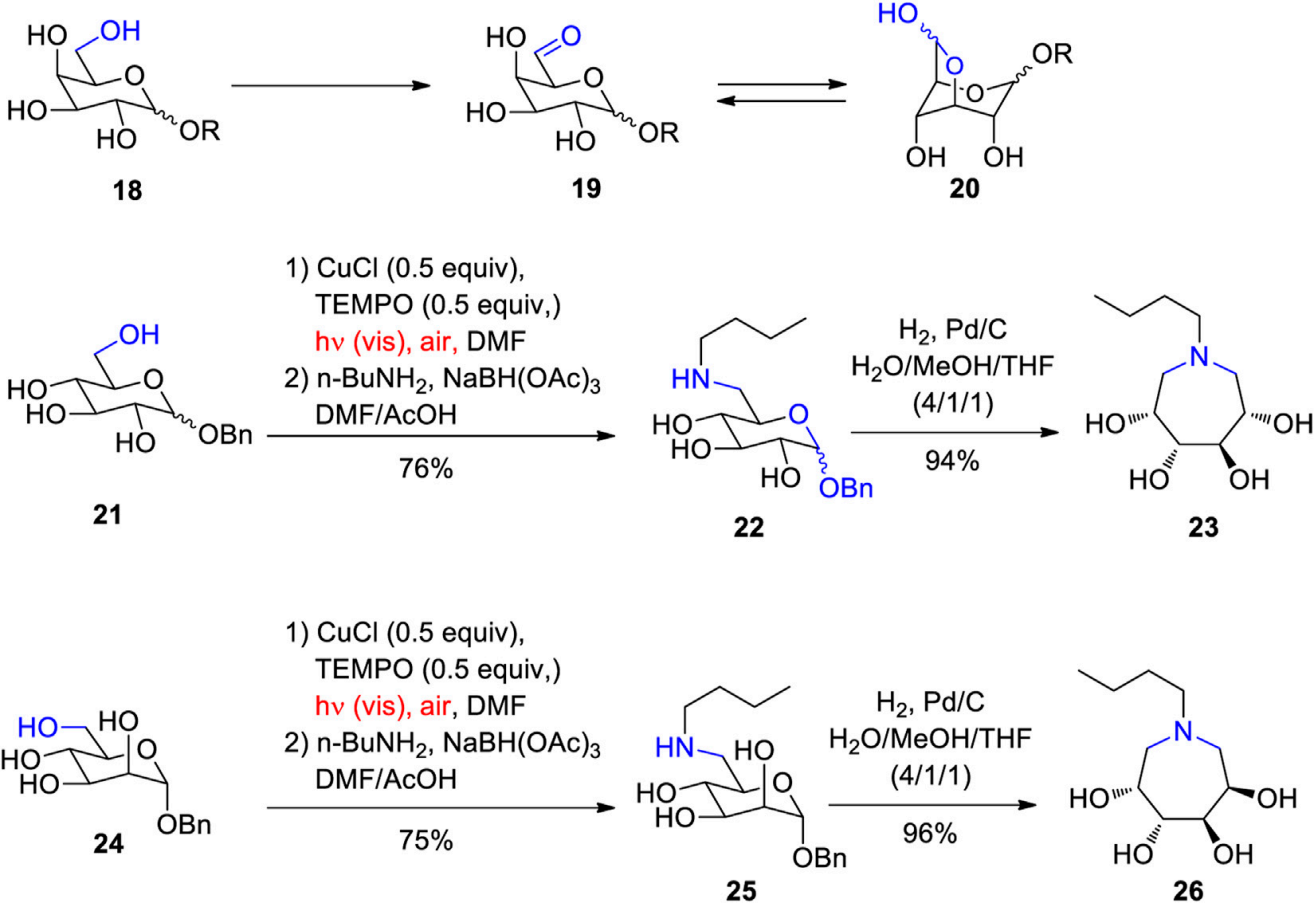
Synthesis of tetrahydroxyazepanes form glucose and mannose derivatives imitating galactose oxidase.

**SCHEME 6:**
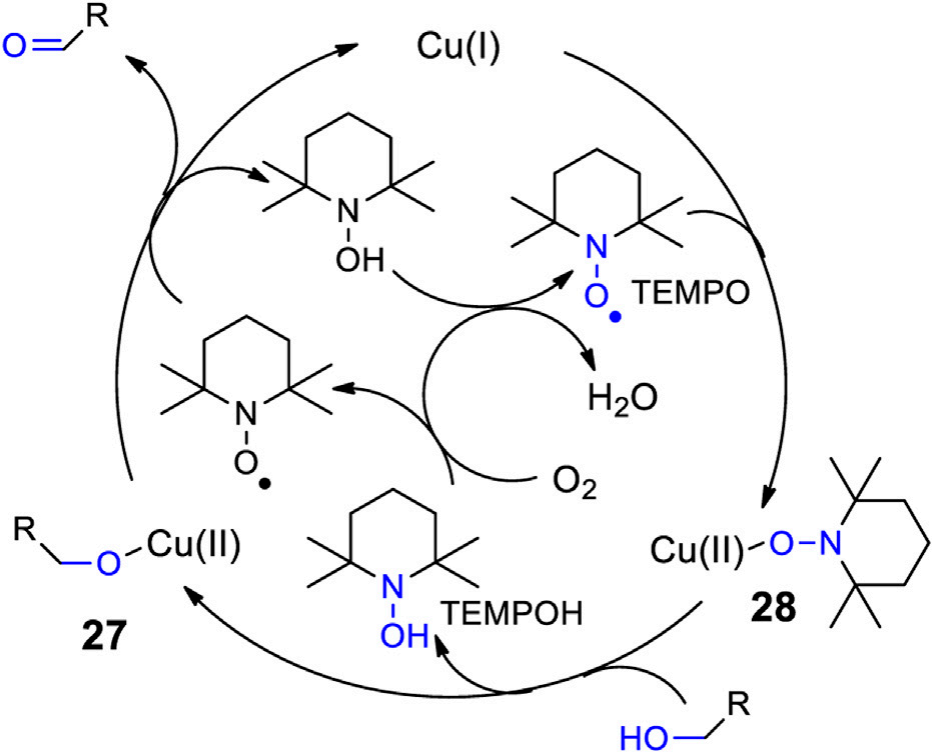
Mechanism of the Semmelhack reaction.

Sugars are considered as platform chemicals when they can be transformed in only few steps into interesting target molecules (Jäger and Minnaard, 2016). Due to the presence of numerous hydroxyl functions in these compounds, selective transformations often need a laborious strategy using protecting groups (Levy and Fügedi, 2006; Kocieński, 2005; Wuts, 2014). In this context, methods are required that enables a selective transformation, for example, of a single hydroxyl function into reactive a carbonyl group. A lot of enzymes enable such transformations. Thus galactose oxidase catalyzes the selective oxidation of the hydroxyl function in the position 6 of galactose into the corresponding aldehyde (Ito et al., 1991). Also artificial catalysts have been developed to imitate such enzyme activities in the context of biomimetic transformations (Pierre, 2000; Chaudhuri et al., 1999; Berkessel et al., 2005; Thomas, 2007; Lewis and Tolman, 2004; Mirica et al., 2004). Such an example is depicted in Scheme 5 (Gassama and Hoffmann, 2008). The galactose oxidase catalyzes the oxidation of compounds like **18** into the corresponding aldehydes **19**. Such products are in equilibrium with their half acetale forms **20** which stabilizes these derivatives and consecutive transformations can be envisaged. In the present case, the oxidation has been carried out using Semmelhack reaction conditions (Semmelhack et al., 1984) with CuCl and TEMPO (2,2,6,6-Tetramethylpiperidinyloxyl) as catalysts and air as oxidant. These conditions are suitable for the oxidation of primary alcohols (Ryland and Stahl, 2014). Under the reported reaction conditions, the oxidation of the compounds such as **21** or **24** was inefficient. However, when carried out under irradiation with visible light, the reaction became efficient. A further improvement was achieved when the reaction mixture was subjected to a reductive amination. The resulting compounds **22** and **25** after deprotection and reductive amination yielded the azepane derivatives **23** and **26**. Such compounds possess interesting pharmaceutical activities (Compain and Martin, 2007; Li et al., 2009; Désiré et al., 2014). As the examples show, this strategy for the synthesis of azepanes can be applied to a larger variety of hexoses with different relative and absolute configuration while a corresponding application of enzymes such as the galactose oxidase is limited to particular stereoisomers. The mechanism depicted in Scheme 6 has been suggested in which Cu(II) acts as the oxidant of the alcohol species (**27**) (Dijksman et al., 2003). The resulting Cu(I) is reoxidized to Cu(II) by addition of TEMPO (**28**) and the release of TEMPOH. The positive effect of irradiation with visible light can be explained by the fact that the Cu-O bond is weakened when such complexes are electronically excited via a ligand to metal charge transfer (LMCT). Thus ligand exchange steps in the mechanism are accelerated (Abderrazak et al., 2021).

**SCHEME 7:**
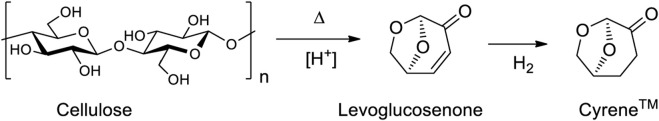
Levoglucosenone as an intermediate in the production of Cyrene™.

Recently levoglucosenone became an interesting platform chemical (Camp and Greatrex, 2022). It is obtained from cellulose by pyrolysis under acidic conditions (Scheme 7) (De bruyn et al., 2016; Halpern et al., 1973; He et al., 2017; Klepp et al., 2020). This compound is now produced on industrial scale as an intermediate in the production of Cyrene™, a biobased aprotic dipolar solvent (Sherwood et al., 2014; Citarella et al., 2022). A relatively high number of functional groups are located on a small enantiopure compound. Thus levoglucosenone is an interesting synthon for asymmetric synthesis (Comba et al., 2018; Awad et al., 2006; Sarotti et al., 2012; Tsai et al., 2018). It can also be transformed into other platform chemicals such as furanones (Diot-Néant et al., 2018; Bonneau et al., 2018). Also Cyrene™, is used as synthon in organic synthesis (Stini et al., 2022).

**SCHEME 8:**
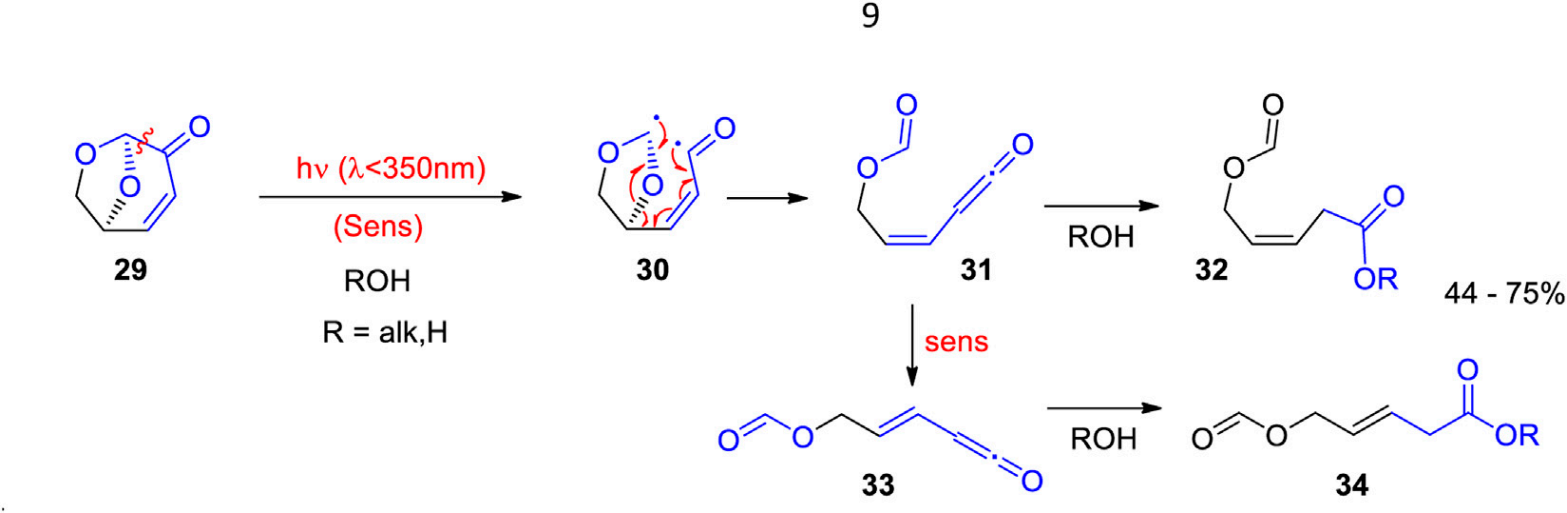
The photochemical reactivity of levoglucosenone is dominated by a Norrish type I reaction.

Hitherto, only few photochemical reactions with levoglucosenone have been reported. When levoglucosenone **29** is electronically excited by light absorption, a Norrish type I reaction occurs yielding the diradical intermediate **30** (Scheme 8) (Yamada and Matsumoto, 1992). Rearrangement yields the ketene **31** which is trapped by an alcohol leading to the corresponding ester **32**. Trans-substituted alkenes **33** and **34** are also formed, most probably via sensitization. Similar steps are often observed in Norrish type I reactions (Bohne, 1995; Majhi, 2021). In this transformation, alkenes with an interesting substitution pattern are obtained.

**SCHEME 9:**
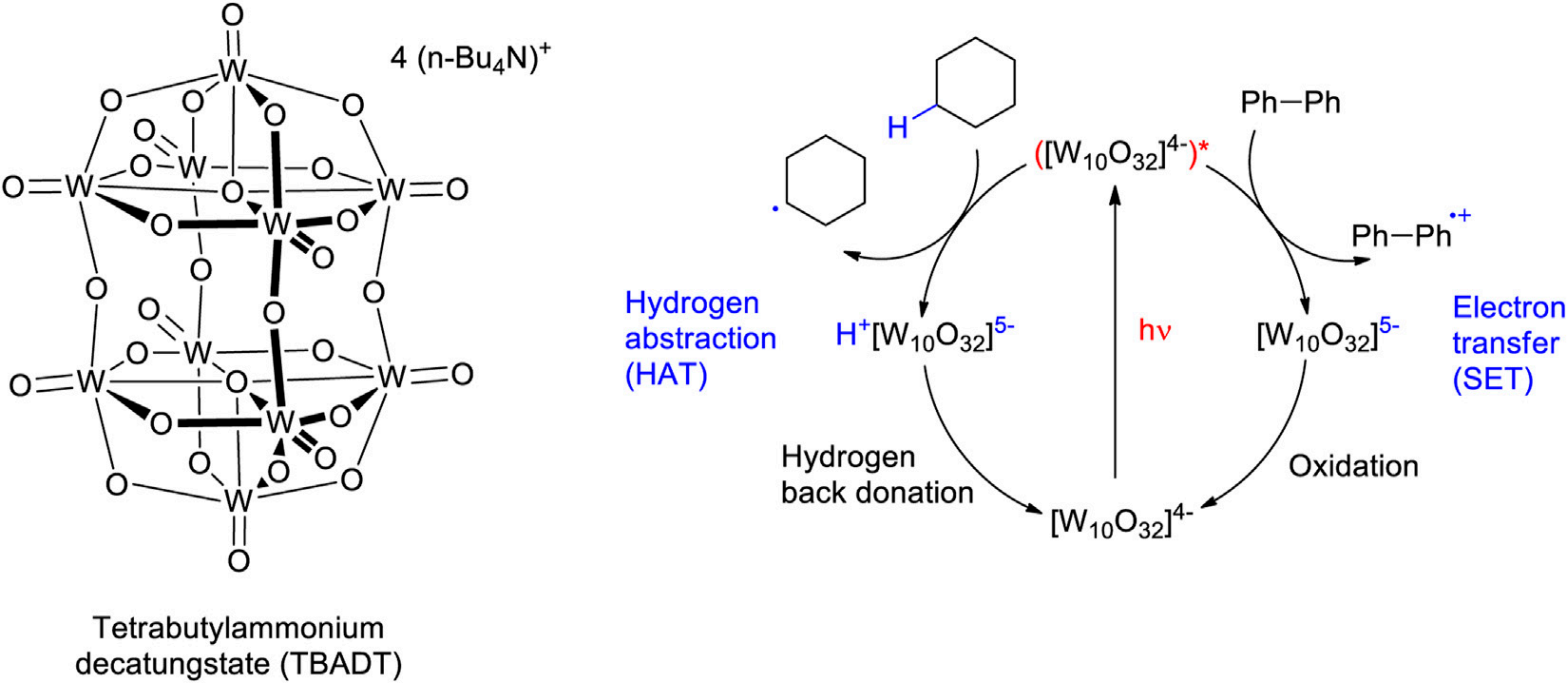
Photocatalytic reactions with tetrabutylammonium decatungstate (TBADT).

However, the complex bicyclic structure is destroyed and the chiral information is lost. In this context photocatalysis with light of longer wavelengths enables photochemical transformations. Under such reaction conditions, a catalytic system absorbs light while the substrate remains at its ground state. Tungstates such as tetrabutylammonium decatungstate (TBADT) are capable of generating radical species (Scheme 9) (Tanielian, 1998; Ravelli et al., 2016; Hong and Indurmuddam, 2024). Such intermediates can be generated by hydrogen atom transfer (HAT) in different ways. In the present context, two processes are often discussed: (a) The proton and the electron are transferred simultaneously or (b) the electron is transferred first and the proton follows (Hoffmann, 2016; Hoffmann, 2015). In a more general context, these processes are part of proton-coupled electron transfer (PCET) (Hoffmann, 2017; Miller et al., 2016; Murray et al., 2022; Tyburski et al., 2021). Also single electron transfer (SET) is observed with these catalysts. The reaction conditions are particularly mild so that also complex polyfunctional substrates such as morphine derivatives can be selectively transformed (Gorbachev et al., 2022).

**SCHEME 10:**
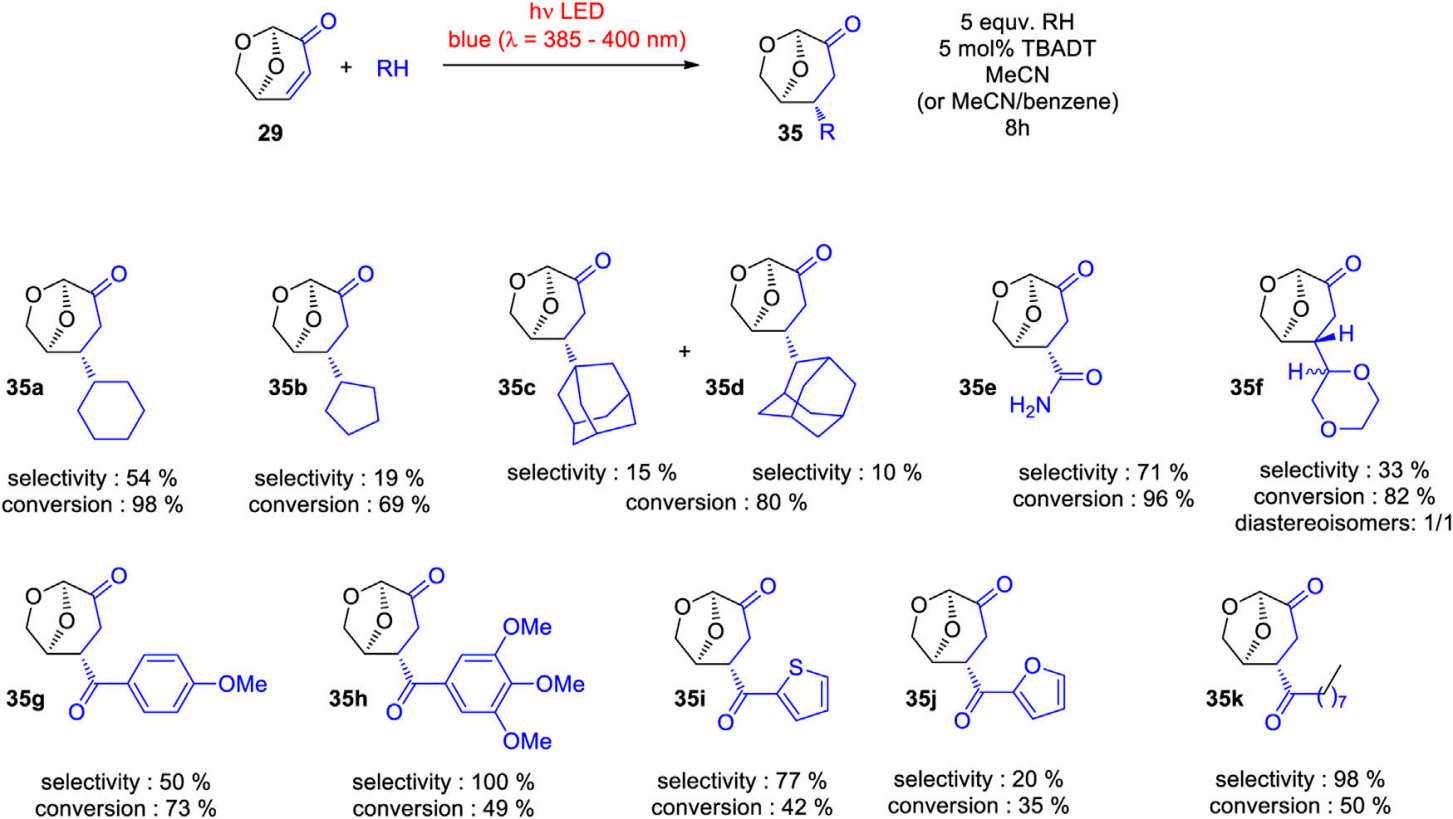
Addition of a variety of photochemically generated radicals to levoglucosenone **29**.

**SCHEME 11:**
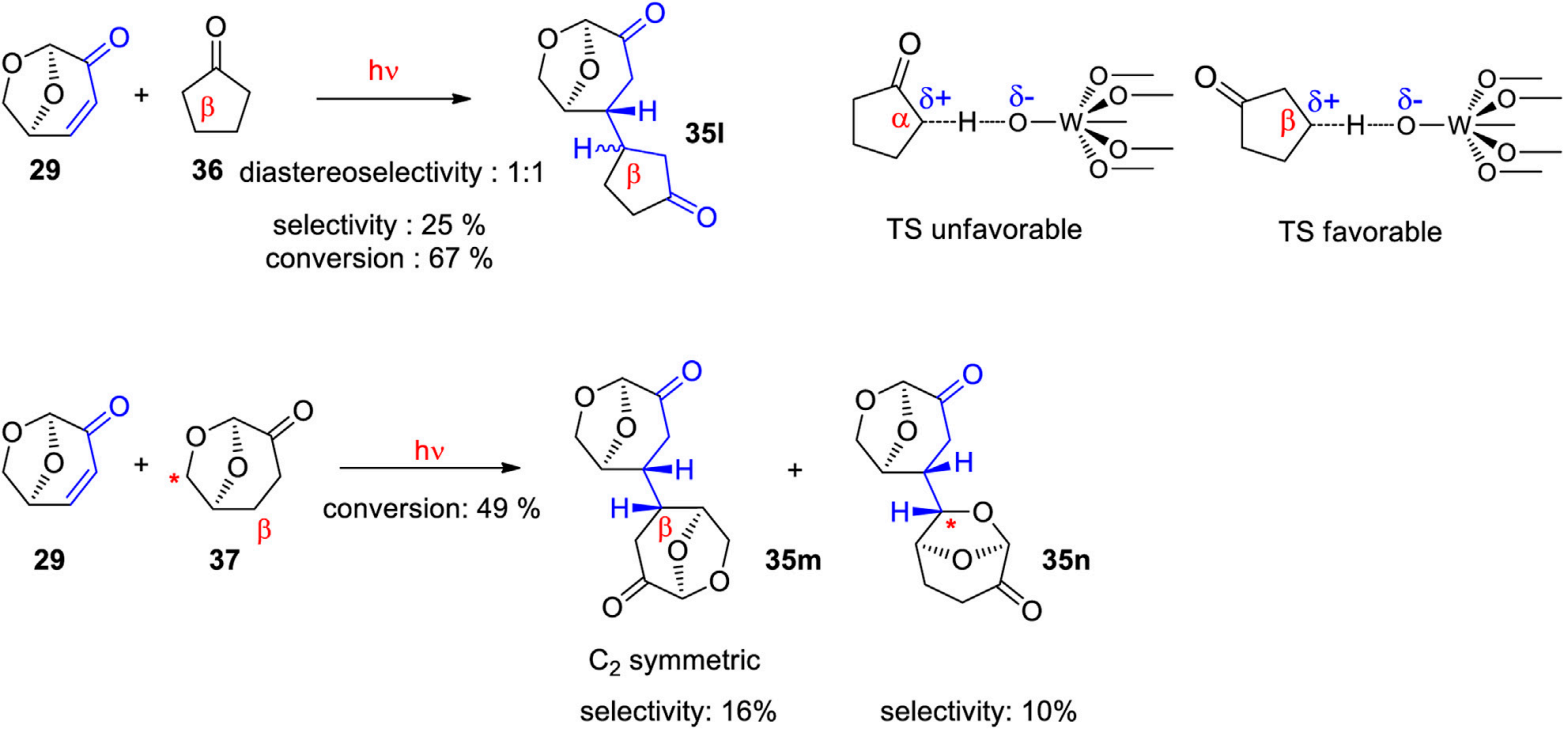
Unusual regioselectivity in the reaction of cyclopentanone **36** to levoglucosenone **29**. Addition of cyrene™ **37** to levoglucosenone **29**.

**SCHEME 12:**
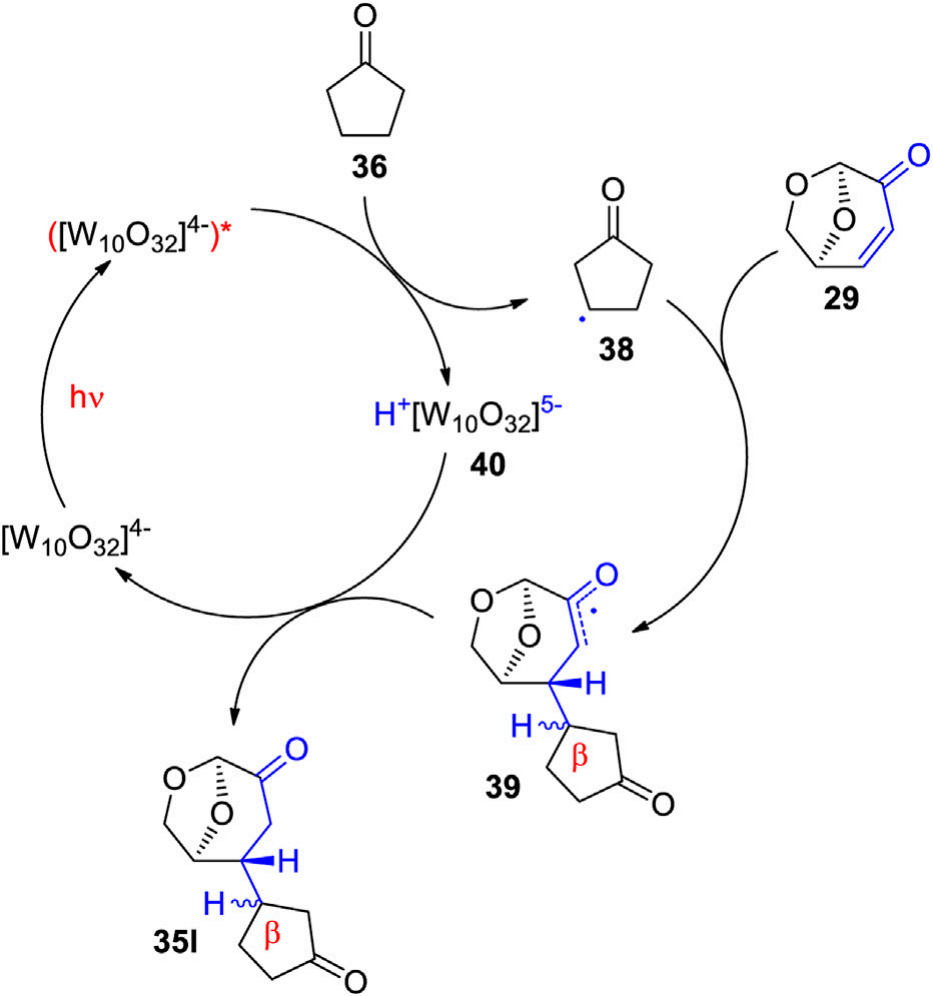
Mechanism of the TBADT photocatalyzed addition cyclopentanone **36** to levoglucosenone **29**.

Using TBADT as photocatalyst, a variety of radical species were generated and added to levoglucosenone **29** (Scheme 10) (Lefebvre et al., 2022). Thus adducts with alkanes (**35a**, **35b**, **35c** or **35d**) have been obtained. The addition of formamidyl radicals (**35e**) was particularly efficient. Cyclic ethers (**35f**) have also been added. A large number of acyl radicals generated from corresponding aldehyde precursors have been added. Adducts with benzaldehyde derivatives (**35g** and **35h**) have been synthesized. Also heterocyclic aldehydes (**35i** or **35j**) and aliphatic aldehydes (**35k**) have been added. The photosensitization with TBADT of the radical addition is generally efficient in the transformation of aromatic aldehydes (Raviola et al., 2019; Qiao et al., 2022). Some recent works particularly deals with reactions of furfural (Nielsen et al., 2024). The radical addition occurred stereospecifically anti with respect to the (CH_2_-O)-bridge in levoglucosenone. An energy difference of the transition states for both diastereotopic attacks of the radical intermediates of 5 kcal∙mol^−1^ was calculated. The high stereoselectivity qualifies the reaction for application to asymmetric synthesis. The particular regioselectivity of the radical addition was observed in the case of cyclopentanone **36** (Scheme 11) (Lefebvre et al., 2022). One should expect the formation of a radical species in the α position of the cyclic ketone due to an increased stability by a mesomeric effect in the resulting intermediate. However, the reaction took place in the β position yielding adduct **35l**. This observation has been explained by the fact that at the transition state (TS) of the hydrogen atom transfer (HAT) step, a positive partial charge is generated at the hydrogen donor partner. In the case of cyclopentanone **36** this is favorable for a reaction in the β position. Detailed investigations of the stereo- and regioelectronic effects in this step have been carried out (Okada et al., 2014; Yamada et al., 2017; Ravelli et al., 2018; Roberts, 1999). The regio and stereoselectivity of such reactions are very high. In the addition of cyrene™ **37**, only two isomers of 32 possible products have been isolated. The hydrogen atom transfer from the β-position of **37**, yields the highly symmetric adduct **35m**. The reaction at the (CH_2_-O)-bridge of **37** yields the adduct **35n**. The reaction mechanism for the addition of cyclopentanone is presented in Scheme 12. After excitation of the decatungstate, a hydrogen atom is transferred from the cyclopentanone to the photocatalyst yielding the radial intermediate **38**. After addition of the latter to levoglucosenone **29**, the electrophilic oxoallyl radical **39** is formed. In the final step, a hydrogen atom is transferred from the reduced photocatalyst species **40** to the intermediate **39** yielding the final product **35l**. In this step, the photocatalyst is regenerated.

## Platform chemicals from lignin

Lignin is an important renewable source of aromatic compounds, especially of phenol derivatives (Farmer et al., 2015; Argyropoulos et al., 2023; Heitner et al., 2010; Zhang and Wang, 2022). However, depolymerization of this complex material for the production of aromatic compounds is challenging (Subbotina et al., 2021; Li et al., 2024; Zhang C. et al., 2023; Zhou et al., 2022). Among other methods, also photochemical, especially photocatalytic reactions are investigated in this context (Li et al., 2016; Zakzeski et al., 2010; Rinaldi et al., 2016; Kärkäs et al., 2016b; Magallanes et al., 2019; Zhang, 2018; Das and König, 2018; Niguyen et al., 2020). Very often, such reactions have been carried out with model compounds. Vanillin is one of the monomers which is currently produced from lignin on the industrial scale (Backa et al., 2012; Bjørsvik and Liguori, 2002; Fache et al., 2016; Araújo et al., 2010; Nayak et al., 2023).

**SCHEME 13:**
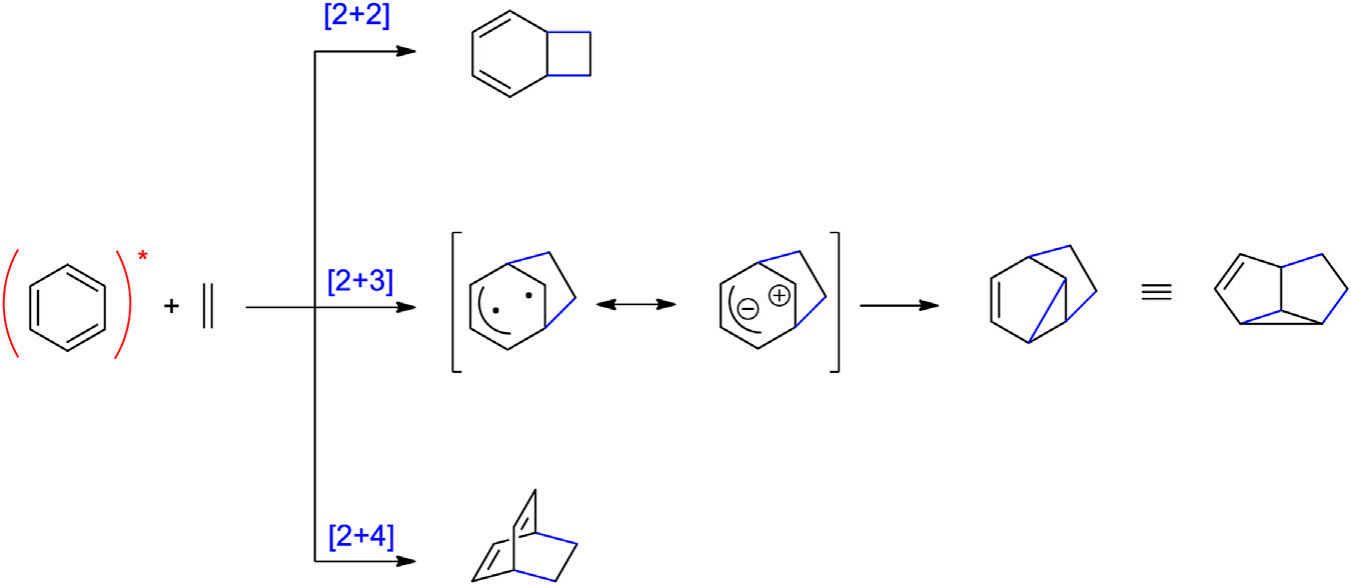
Three types of photocycloadditions of electronically excited benzene derivatives with alkenes.

Concerning photochemical transformations, the reactivity of electronically excited aromatic compounds is significantly different from their ground state reactivity. At the ground state, these compounds possess aromatic character. At the excited state (Franck Conton state) they are anti-aromatic (Rosenberg et al., 2014; Yan et al., 2023). Consequently, they become particularly reactive. In contrast to many ground state reactions, photochemical reactions are characterized by a high tendency to avoid the aromatic stabilization in the final products. This property is particularly interesting for application to organic synthesis since a high degree of molecular complexity is generated in such reactions (Hoffmann et al., 2016). The photochemical cycloadditions of electronically excited aromatic compounds with alkenes are typical examples (Hoffmann, 2012; Remy and Bochet, 2016; Hoffmann, 2004). Generally, three types of such reactions are observed with benzene derivatives (Scheme 13) (Cornelisse, 1993; Cornelisse et al., 2001). The [2+2] and the [2+3] photocycloaddition are often observed as competing reactions. The product ratios often depend on the substitution pattern or the redoxpotentials of the reaction partners (McCullough, 1987; Müller and Mattay, 1993; Desvals and Hoffmann, 2023). While the [2+3] was often applied to the synthesis of complex compounds (Hoffmann, 2012; Remy and Bochet, 2016; Desvals and Hoffmann, 2023; Wender et al., 1990; De Keukeleire and He, 1993; Streit and Bochet, 2011; Hoffmann et al., 2005; Zhang et al., 2020), the [2+2] photocycloaddition is only recently and in systematic way applied to organic synthesis (Gilbert and Bach, 2023; Proessdorf et al., 2022). In the case of the [2+3] photocycloaddition and its application to the synthesis of natural products, it was shown that the efficiency is improved when the reaction is carried out under continuous flow conditions, which open perspectives for large-scale transformations (Alshammari et al., 2024). The [2+4] cycloaddition is less frequently observed in such transformations.

**SCHEME 14:**
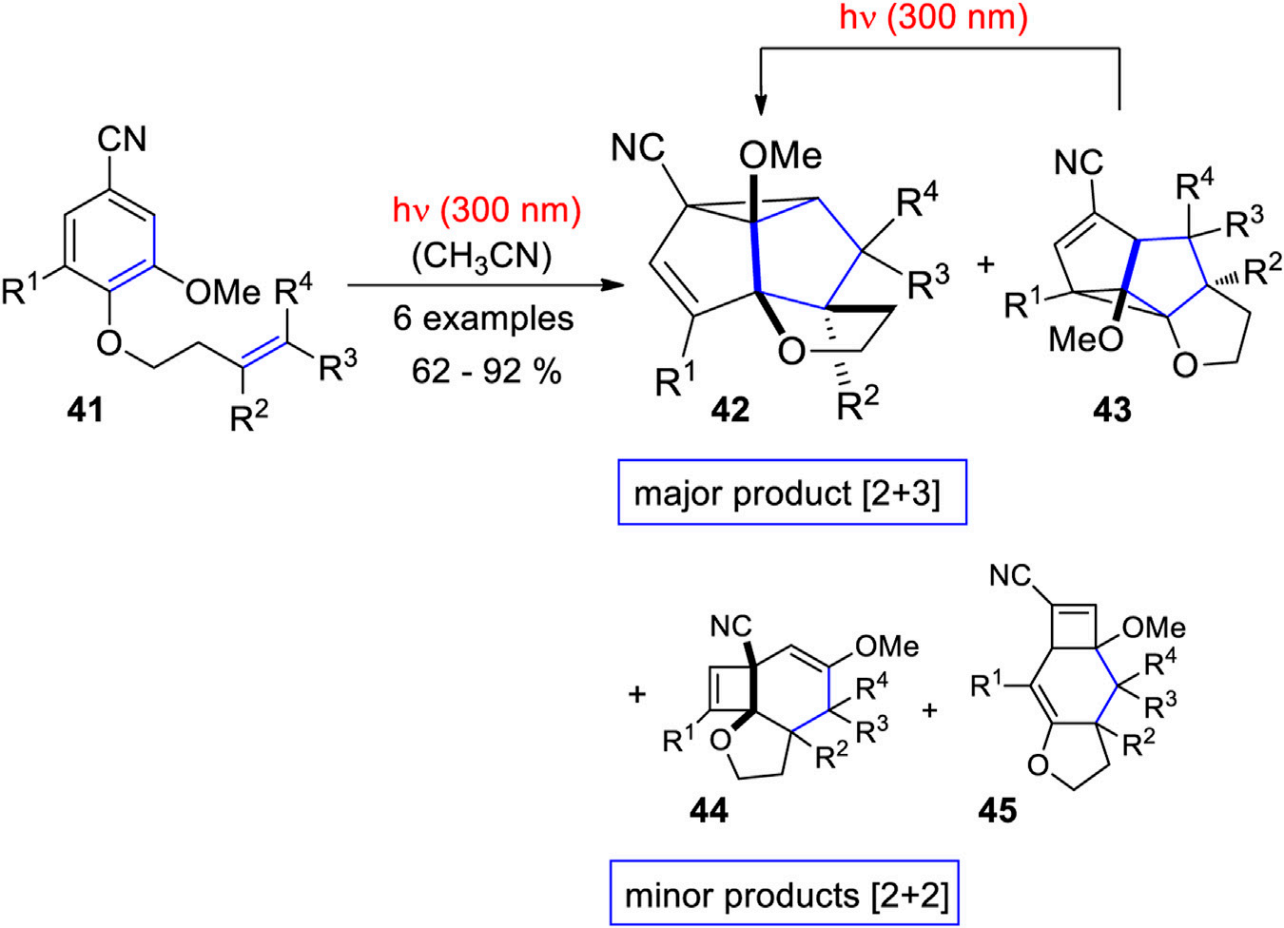
Intramolecular photocycloaddition of vanillin derivatives. Products result from an initial [2+3] or [2+2] photocycloaddition.

**SCHEME 15:**
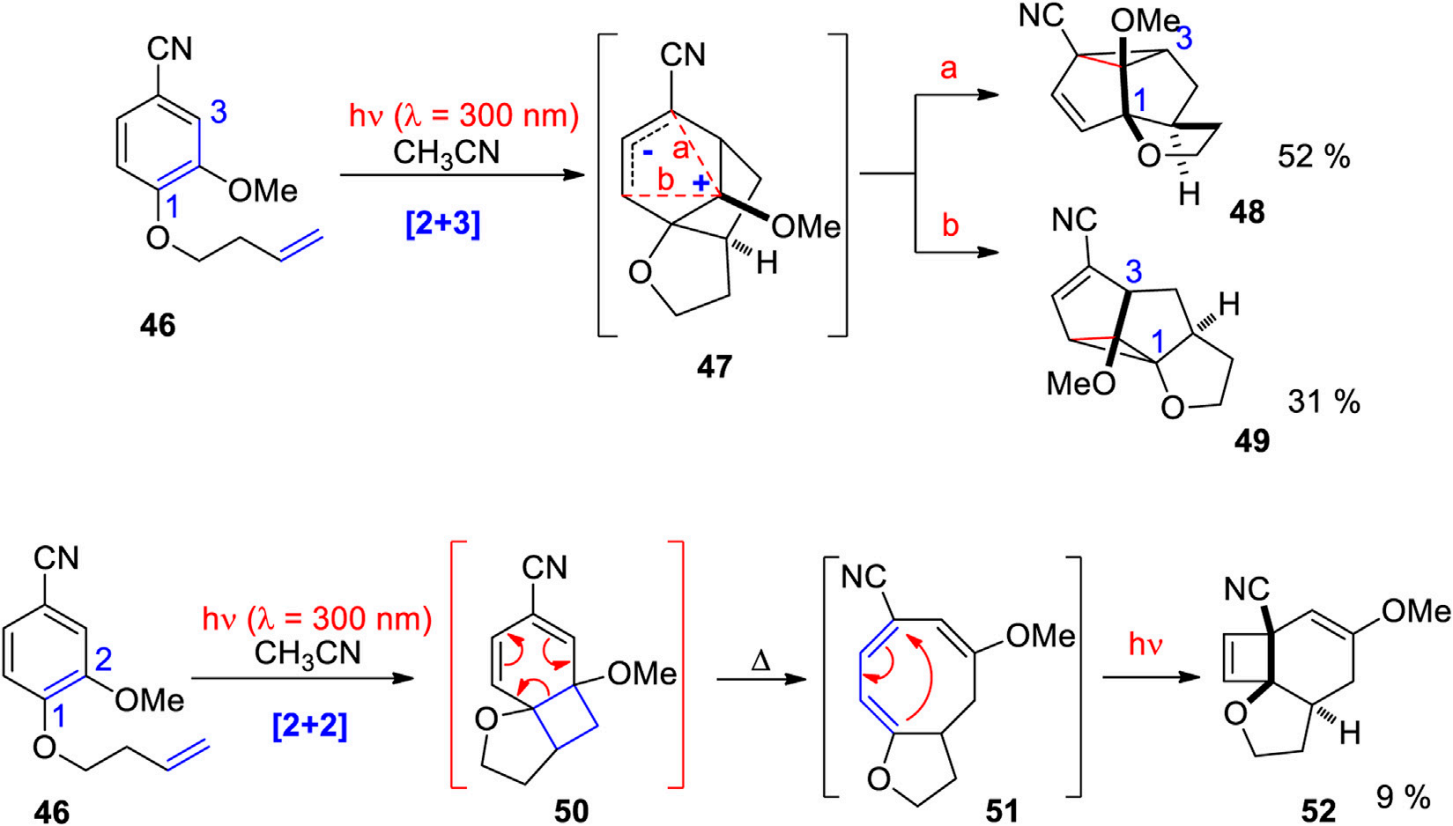
Mechanisms for the formation of complex molecules resulting from initial [2+3] or [2+2] photocycloaddition. These reactions occur at the singlet state.

In this context, intamolecular photocycloadditions of vanillin derivatives have been investigated. When compounds such as **41** – the aldehyde group was transformed into a nitrile function – are irradiated at λ = 300 nm, two types of products are formed (Scheme 14) (Desvals et al., 2021). The linear triquinane derivatives **43** and the corresponding angular derivatives **42** result from a [2+3] cycloaddition while tricyclic cyclobutene compounds **44** and **45** result from an initial [2+2] cycloaddition followed by thermal and photochemical rearrangements. Also, angular (**44**) and linear regioisomers (**45**) for this compound family are formed. The product ratio depends on the substitution pattern. In the case of the [2+3] adducts, the linear isomer **43** absorbs light at λ = 300 nm. Consequently, this compound is transformed into the angular isomer **42**. It was also shown that the product ratio depends on the spin multiplicity of the electronically excited benzene moiety. Reactions depicted in Scheme 14 are singlet processes. Reactions at the triplet state are sensitized transformations in which triplet energy is transferred to the aromatic substrate. These reactions are less efficient and different isomers only resulting from initial [2+2] photocycloaddition followed by thermal and photochemical rearrangements are isolated. Reaction mechanisms of the singlet reactions of vanillin derivatives are depicted in Scheme 15. In the case of the main products resulting from a [2+3] cycloaddition, the alkene is added at the 1,3 positions of the photochemically excited benzene moiety and the intermediate **47** is generated. Due to the singlet multiplicity and the presence of polar substituents – the methoxy and the cyano group – this intermediate possesses zwitterionic character. Charge combination may occur in two ways. Path a yields the angular isomer **48** and path b generates the linear isomer **49**. In the case of an initial [2+2] photocycloaddition in positions 1 and 2, the primary adduct **50** undergoes electrocyclic ring opening in a thermal disrotatory process involving 6 electrons and yielding the cyclooctatriene intermediate **51**. A photochemical disrotarory involving 4 electrons yields the final product **52**. In this case, only the angular isomer is generated. It must be pointed out that such pericyclic reactions steps are reversible. In the case of photochemical reactions, often photostationary equilibria are involved. For example, primary [2+2] adducts such as **50** also absorbs light and cycloreversion or retrocycloaddition may become efficient. In these cases, no photochemical conversion is observed under standard conditions. Primary photocycloadducts can be trapped, for example, by an acid catalyzed reaction and the photostationary equilibrium is displaced towards the product site and photoproducts can be isolated (Hoffmann and Pete, 1995; Hoffmann and Pete, 1997; Hoffmann et al., 2002; Hoffmann, 2002). Such conditions extend the scope of these reactions and further application to organic synthesis are envisaged. For example, rigidified dopamine analogues (Verrat et al., 2000; Verrat, 2000) or compounds possessing the of 5,5-dialkylcyclohexane-1,3-dione core structure of a herbicide family (Hoffmann and Pete, 2001) have been synthesized with this reactions as a key step.

Photochemical reactions have been carried out also with hydrazones or oximes and related compounds derived from aromatic aldehydes (Latrache and Hoffmann, 2021). Thus oximes of vanillin and related compounds derived from lignin are transformed into corresponding oximes. Under photochemical conditions, they are transformed into nitriles (Ban et al., 2019; Joy et al., 2022). Condensation of vanillin and or syringaldehyde with Meldrum’s acid yields UV-A and blue light filters (Peyrot et al., 2020). Such compounds possessing a phenol moiety have also radical trapping properties. Therefore, they are particularly safe compared to established sunscreen compounds.

**SCHEME 16:**
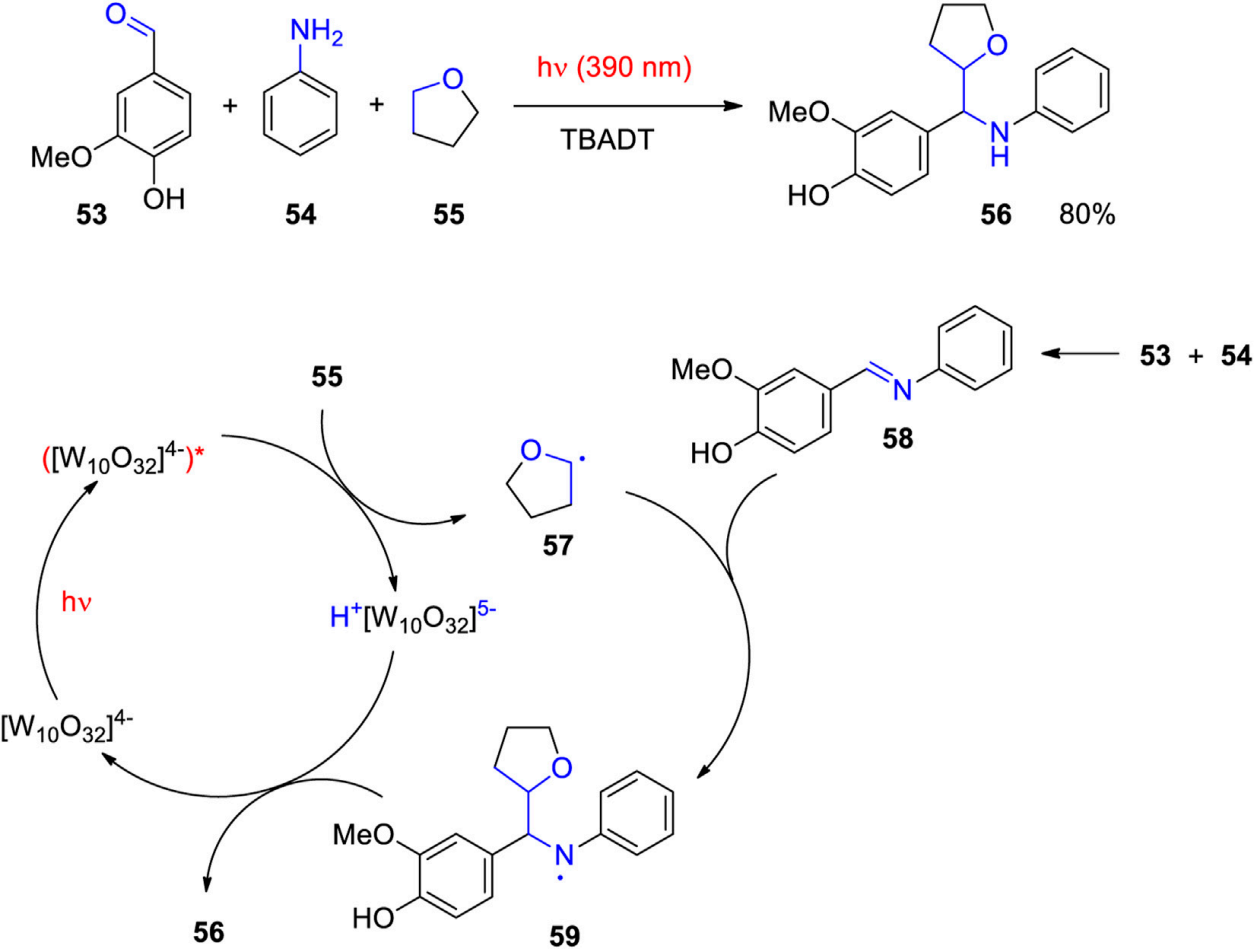
Photocatalytic radical addition to an imine (**58**) as key step in a three component reaction with vanillin **53**.

Aromatic aldehydes are suitable synthons for organic synthesis. An enormous number of syntheses with these compounds are reported, among them multi component reactions (Dömling et al., 2012; Nazeri et al., 2020). In a three component photocatalyzed reaction, vanillin **53** reacts with aniline **54** and tetrahydrofurane (THF) **55** yielding compound **56** (Scheme 16) (Pillitteri et al., 2021). As previously explained radical intermediates **57** are generated from THF **55** using photocatalysis with TBADT (Tanielian, 1998; Ravelli et al., 2016; Hong and Indurmuddam, 2024). Imine intermediates are formed by condensation of vanillin **53** with aniline **54**. The alkyl radical **57** selectively adds to the imine **58** leading to the intermediate **59**. The latter is reduced by hydrogen atom transfer from the photocatalyst. In this step, the final product **56** is formed and the catalyst is regenerated.

## Platform chemicals from biotechnology

Most of the biomass contains oxygen rich compounds. On the other hand, traditionally chemical industry uses huge quantities of oxygen-free compounds such as alkenes as platform chemicals. Although several compounds, for example, terpenes or natural rubber are synthesized by plants, biotechnological processes are developed to produce alkenes on large scale (Lee et al., 2019; Wilson et al., 2018; van Leeuwen et al., 2012; Saaret et al., 2021). The transformation of oxygen rich biomass derived platform chemicals to alkenes is systematically studied (Nakagawa et al., 2023).

**FIGURE 1:**
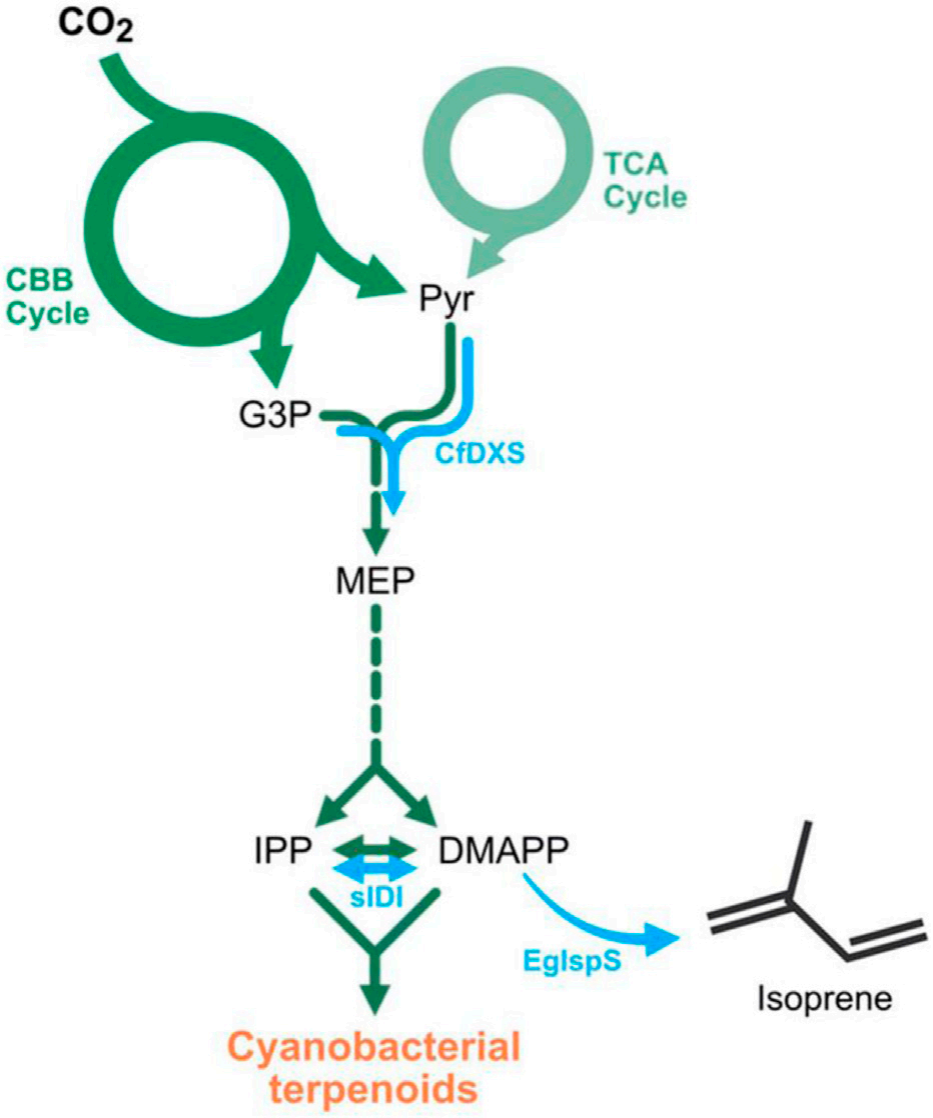
Cyanobacterial terpenoid pathway (green) and genetic modifications favoring the production of isoprene (blue). CBB, Calvin–Benson–Bassham; TCA, tricarboxylic acid; Pyr, pyruvate; G3P, glyceraldehyde-3-phosphate; MEP, methylerythritol-4-phosphate; IPP, isopentenyl-pyrophosphate; DMAPP, dimethylallyl-pyrophosphate; CfDXS, 1-deoxy-d-xylulose-5-phosphate synthase from Coleus *forskohlii*; sIDI, IPP/DMAPP isomerase from *Synechocystis* sp. PCC 6803; EgIspS, isoprene synthase from Eucalyptus globulus [Adapted form ref. Rana et al. (2022)].

**SCHEME 17:**
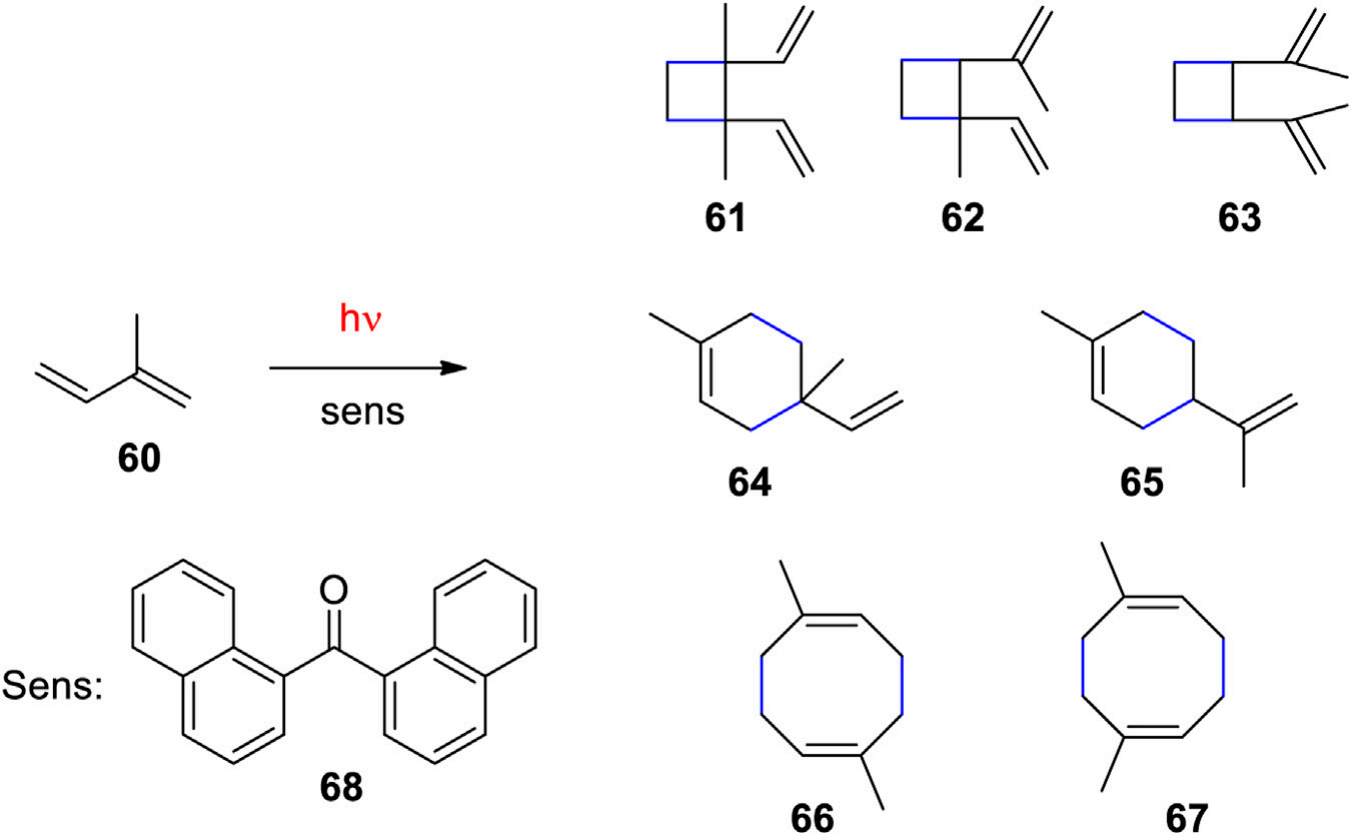
Photosensitized dimerization of isoprene **60** using triplet sensitization with the dinaphthylketon **68**.

Recently, a process was developed for the production of C_10_ cycloalkanes which fulfill requirements of jet fuels (Rana et al., 2022). A photobiological transformation is followed by a photochemical reaction. Using photosynthesis, the cyanobacterium *Synechocystis* transforms CO_2_ into terpenes. In order to favor the production of isoprene, genetic modifications have been carried out (Figure 1). Such methods enable the non-farming production of biomass. Photosensitized dimerization of isoprene **60** yielded a variety of [2+2] **61**, **62** and **63**, [2+4] **64** and **65** and [4+4] cycloadducts **66** and **67** (Scheme 17) (Rana et al., 2022). The reaction was first carried out with benzophenone as sensitizer (Hammond et al., 1963). It was found that the product ratio depends on the triplet energy of the sensitizer (Liu et al., 1965). In the present study, the reaction was further optimized by using the dinaphthylketone **68** as sensitizer. Various other ketones were less efficient. Although, this ketone absorbs light close to the visible domain, its triplet energy is still high enough to excite isoprene **60** to the triplet state by energy transfer. Using a particular setup in which the mixture of isoprene **60** and the sensitizer **68** (0.1 mol%) was kept in a sealed fluorinated ethylene propylene tube, cooled to ∼10°C and irradiated at (λ = 365 nm) the mixture of dimers was obtained with 89% yield (120 ml scale, product quantum yield Φ = 0.91). The reaction was also carried out with solar irradiation or irradiation with a sunlight simulator. A detailed computational investigation of the reaction mechanism was carried out (Vajravel et al., 2023). In order to get the jet fuel compounds, the product mixture of the photoreaction was hydrogenated using Pd/C as catalyst. Similar reaction conditions have been studied for the photodimerization and cross dimerization of various terpenes (Cid Gomes et al., 2023). The [2+2] photocycloaddition as key step for the production of jet fuels was also studied with furfural derived compounds (Lebedeva et al., 2024) or with terpenes (Xie et al., 2019). It should be pointed out the present process as a combination of photobiological and a photochemical transformation perfectly corresponds to the requirement of a sustainable chemical industry as discussed by Ciamician (1912), Ciamician (1908) and others more than 100 years ago. This event can be considered as the beginning of green or sustainable chemistry (Albini and Fagnoni, 2004; Albini and Fagnoni, 2008).

## Conclusion

Platform chemicals play a central role in chemical industry. In the context of sustainable chemistry, new concepts of their production and transformation are essential. In this context, photochemical reactions play an important role. Existing or new platforms can be produced from biomass as renewable feedstock. As biomass possesses particular structure elements that are less common in fossil feedstock based platform compounds, this material offers numerous accesses to new innovative starting compounds for many domains of chemical industry. Thus carbohydrate based, oxygen rich biomass is transformed into furans. These heterocyclic aromatic compounds are used as starting compounds in many syntheses. The photooxygenation of furans yields interesting synthesis intermediates that are themselves suitable platform chemicals. Furthermore, photooxygenation of furans can easily be carried out on the industustrial scale or on large scale using sunlight as renewable energy source. Recently, an efficient process for the industrial production of levoglucosenone by pyrolysis from cellulose containing biomass has been developed. This compound is an intermediate for the mass production of the agro-solvent cyrene™. Photochemical or photocatalytic transformations of this compound open new perspectives for the valorization of this compound in the chemical or pharmaceutical industry. Carbohydrates such as hexoses or pentoses can also more directly be transformed, for instance, into heterocyclic targets. Photochemical reactions play a key role in sustainable chemistry and in organic synthesis. They enable the access to compounds that are not or difficultly available with more conventional methods of organic synthesis. Many original transformations can be carried out without chemical activation and the photon is considered as a traceless reagent. A more consequent application of these reaction conditions to the transformation of biomass derived platform chemicals efficiently contributes to a sustainable chemical industry as it was described by G. Ciamician more than hundred years ago. In this regard recently, fermentation processes based on the photosynthesis have been developed for the industrial production of alkenes such as isoprene. Using photochemical reactions of these compounds or several other terpenes for the further production of targets represents a very innovative concept for a sustainable industry as it has been shown for the production of jet fuels.
